# Performance of Generative Artificial Intelligence in Dental Licensing Examinations

**DOI:** 10.1016/j.identj.2023.12.007

**Published:** 2024-01-19

**Authors:** Reinhard Chun Wang Chau, Khaing Myat Thu, Ollie Yiru Yu, Richard Tai-Chiu Hsung, Edward Chin Man Lo, Walter Yu Hang Lam

**Affiliations:** a Faculty of Dentistry, The University of Hong Kong, Hong Kong Special Administrative Region, China; b Department of Computer Science, Hong Kong Chu Hai College, Hong Kong Special Administrative Region, China; c Musketeers Foundation Institute of Data Science, The University of Hong Kong, Hong Kong Special Administrative Region, China

**Keywords:** Artificial intelligence, Communication, Dental education, Digital technology, Examination questions, Specialties, Dental

## Abstract

**Objectives:**

Generative artificial intelligence (GenAI), including large language models (LLMs), has vast potential applications in health care and education. However, it is unclear how proficient LLMs are in interpreting written input and providing accurate answers in dentistry. This study aims to investigate the accuracy of GenAI in answering questions from dental licensing examinations.

**Methods:**

A total of 1461 multiple-choice questions from question books for the US and the UK dental licensing examinations were input into 2 versions of ChatGPT 3.5 and 4.0. The passing rates of the US and UK dental examinations were 75.0% and 50.0%, respectively. The performance of the 2 versions of GenAI in individual examinations and dental subjects was analysed and compared.

**Results:**

ChatGPT 3.5 correctly answered 68.3% (n = 509) and 43.3% (n = 296) of questions from the US and UK dental licensing examinations, respectively. The scores for ChatGPT 4.0 were 80.7% (n = 601) and 62.7% (n = 429), respectively. ChatGPT 4.0 passed both written dental licensing examinations, whilst ChatGPT 3.5 failed. ChatGPT 4.0 answered 327 more questions correctly and 102 incorrectly compared to ChatGPT 3.5 when comparing the 2 versions.

**Conclusions:**

The newer version of GenAI has shown good proficiency in answering multiple-choice questions from dental licensing examinations. Whilst the more recent version of GenAI generally performed better, this observation may not hold true in all scenarios, and further improvements are necessary. The use of GenAI in dentistry will have significant implications for dentist–patient communication and the training of dental professionals.

## Introduction

The rapid advancement of artificial intelligence (AI) in recent years has led to significant progress in natural language processing (NLP) and large language models (LLMs).^1^^,^^2^ Amongst these developments, generative AI (GenAI) models, such as ChatGPT (OpenAI), have emerged as sophisticated tools with the ability to comprehend complex conversations and generate humanlike text responses.^3^^,^^4^

Similar to the advent of the internet in the 1990s,^5^ patients may increasingly turn to GenAI for oral health information and guidance. Dental professionals may also use GenAI to answer patients’ inquiries and to facilitate scientific writing and learning.^6^, ^7^, ^8^ However, ensuring the accuracy of the information provided by these AI systems is of utmost importance, given the potential consequences of inaccurate information on patient management and dental education.^5^^,^^9^^,^^10^

Two versions of ChatGPT are available: The older system, ChatGPT 3.5 (GPT-3.5), launched in November 2022, and the latest version, ChatGPT 4.0 (GPT-4), launched in March 2023 and is claimed to have improved performance due to advancements in its algorithm and increased training data. Notably, GPT-4 has more parameters and computational power, which enables it to effectively manage intricate tasks and language patterns and handle a broader range of natural language scenarios.^11^^,^^12^ In the field of health care, ChatGPT is being applied to improve scientific writing, enhance utility in health care research, streamline clinical practice, and provide benefits in health care education.^13^

Existing literature on AI applications in oral health care mainly focusses on diagnostics, treatment planning, and dental treatment.^14^, ^15^, ^16^, ^17^ For example, AI has been used to detect dental caries and periodontal disease from radiographs and photographs and to predict prosthodontic treatment outcomes. However, research investigating the proficiency of GenAI models in dental knowledge, particularly the knowledge that aids in the prevention, diagnosis, and management of oral disease and to promote and maintain oral health,^18^ remains scarce. This highlights a gap in the literature, warranting further investigation into the performance of AI systems in dental knowledge. Given the potential implications of LLMs on patient management and dental education, assessing their accuracy in providing dental information is imperative.

Dental licensure examinations are critical for validating dental graduates' knowledge and competence in providing safe and effective dental care. These examinations establish a benchmark for the desired level of expertise following professional training. This study investigated whether ChatGPT, in either or both versions, can process proficient dental knowledge on par with validated dental graduates and compares the results between the 2 versions. The research hypothesis posits that ChatGPT can achieve performance levels in dental licensing examinations equivalent to validated dental graduates.

## Methods

### Selection of dental knowledge questions

The US and the UK have the highest proportion of the top 100 dental schools according to the latest Quacquarelli Symonds ranking.^19^ This indicates that the dental programmes of these 2 countries meet global standards, and their dental licensing examinations can serve as a benchmark for dental knowledge. Moreover, these examinations are amongst the most popular worldwide, with numerous sample questions available. They cover various dental subjects, including oral surgery, orthodontics, periodontics, and prosthodontics, ensuring a comprehensive evaluation of the subject matter. The Integrated National Board Dental Examination (INBDE)^20^ and the Overseas Registration Examination (ORE)^21^ are the US and the UK dental licensing examinations, respectively, and were selected as the sampling base for this study.

The questions for this study were derived from examination preparation books for the INBDE and ORE. The top relevant seller for NBDE Book on the US online bookstore Amazon was selected for INBDE.^22^ The only ORE series on the UK online bookstore Book Depository^23^ was chosen for ORE.

Of all multiple-choice questions found in the INBDE^24^ and the ORE books^25^^,^^26^ were included in this study, except those that contained figures or tables. This was due to limitations in inputting these graphical or nontext elements into the ChatGPT system.

### Input into GenAI

The questions were input into GPT-3.5 by an independent assessor (RC) in the exact format as they appeared in the books between April 21, 2023, and April 23, 2023 (Figure). Similarly, the same questions were then meticulously inputted into GPT-4 by the same assessor, ensuring that the format, wording, and punctuation remained identical. Input for GPT-4 commenced between May 8, 2023, and May 23, 2023.

**Fig:**
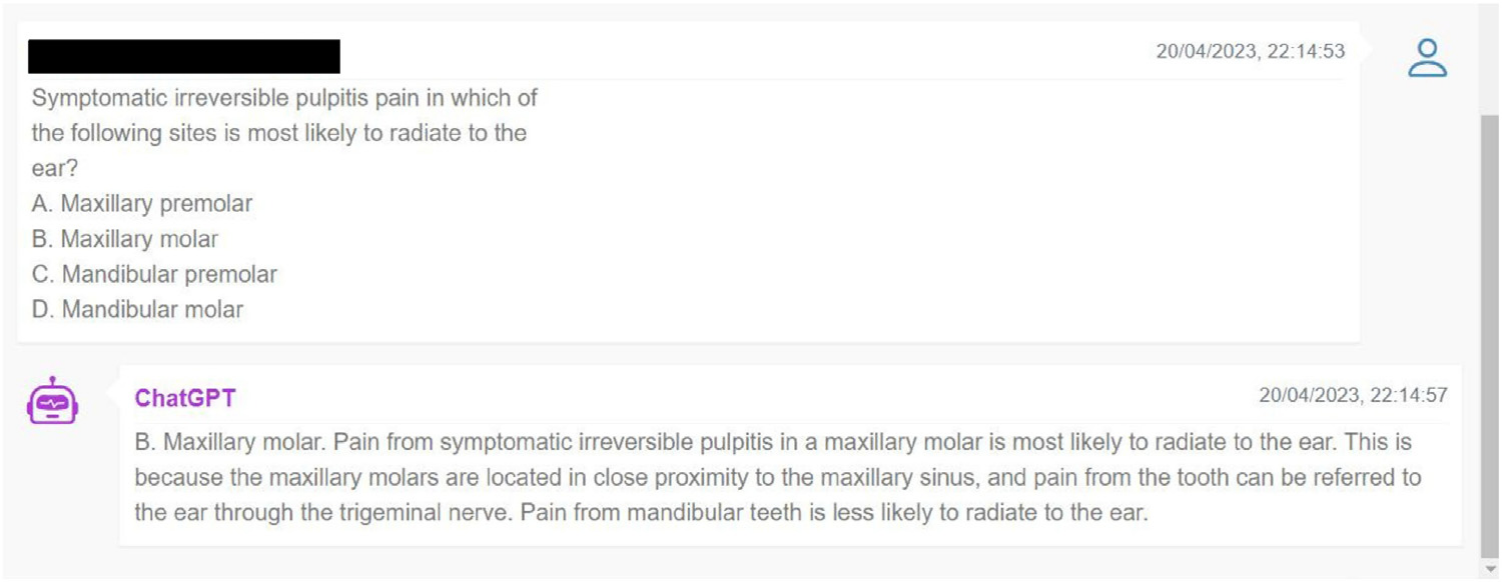
Illustration of a live input into a chatbot (above), with ChatGPT providing its response. (below)

### Analysis of GenAI performance

Once the questions were entered into the system, the multiple-choice answers selected by ChatGPT were collected, and the suggested answer keys provided in the selected books were used as the gold standard to compare the AI system's performance. The passing rates of the US and UK dental examinations were 75.0% and 50.0%, respectively. Moreover, an analysis was conducted on the AI system's performance in individual dental subject areas, and the numbers of correct and incorrect answers were meticulously recorded.

The results of GPT-3.5 and GPT-4, including the number of correct and incorrect answers and the performance of individual dental subjects, were then compared. This comparison highlights any improvements or differences in the AI's performance between the 2 versions. It serves as a resource for understanding the evolution and progress of the ChatGPT system's proficiency in oral health knowledge.

## Results

A total of 1461 questions were selected for this study, including 745 questions for the INBDE and 716 questions for the ORE. Thirty-two questions with figures or tables were excluded, all from the ORE textbooks. GPT-3.5 correctly answered 805 out of the 1461 questions, scoring 56.3%; GPT-4 correctly answered 1030 out of the 1429 questions, scoring 72.1%, which was higher than GPT-3.5’s score.

For the 745 INBDE questions, GPT-3.5 correctly answered 509, achieving a score of 68.3%, whereas GPT-4 correctly answered 601, achieving a score of 80.7%, a better result than GPT-3.5. Since the written examination of the INBDE required candidates to score 75.0% or above to pass,^27^ GPT-3.5 failed the examination, whilst GPT-4 passed. The detailed dental subject–specific performance of GPT-3.5 and GPT-4 for the INBDE questions is reported in Table 1.

**Table 1:** Summary of the performance of ChatGPT 3.5 and ChatGPT 4.0 in answering the Integrated National Board Dental Examination (INBDE) questions by dental subjects. Color key: green, ChatGPT 4.0 performed better; red, ChatGPT 3.5 performed better.

Of the 684 ORE questions,^25^^,^^26^ GPT-3.5 answered 296 correctly, scoring 43.3%, whilst GPT-4 answered 429 correctly, scoring 62.7%, an improvement from GPT-3.5. Since the written examination of ORE required candidates to achieve 50.0% or more to pass,^21^ GPT-3.5 failed this examination, whilst GPT-4 passed. The detailed dental subject–specific performance of GPT-3.5 and GPT-4 for the ORE questions is reported in Table 2.

**Table 2:** Summary of the performance of ChatGPT 3.5 and ChatGPT 4.0 in answering the Overseas Registration Examination (ORE) questions by dental subjects. Color key: green, ChatGPT 4.0 performed better; red, ChatGPT 3.5 performed better.

Regarding individual subjects, GPT-4 showed improvement in all dental subjects compared to GPT-3.5, except for the Child Dental Health and Orthodontics in the ORE. However, both versions performed relatively poorly in restorative dentistry/prosthodontics, followed by orthodontics and paediatric dentistry. The detailed comparison of the performance of the 2 ChatGPT models is also reported in Table 1 (INBDE) and Table 2 (ORE), respectively.

There were 49 questions that GPT-3.5 answered correctly but GPT-4 answered wrong for the INBDE. For the ORE, the number was 53. The 2 versions of ChatGPT did not always provide the same correct or incorrect answers to questions. Although GPT-4 demonstrates better performance in answering dental knowledge questions, there were a few instances where GPT-3.5 answered correctly. This phenomenon was observed across all subjects for the INBDE (Table 3) except for periodontics for the ORE (Table 4).

**Table 3:** Numbers of questions that ChatGPT 3.5 answered correctly but ChatGPT 4.0 answered wrong for the Integrated National Board Dental Examination (INBDE) questions by dental subjects.

	No. of questions that ChatGPT 3.5 answered correctly but ChatGPT 4.0 answered wrong
Pharmacology	**2**
Periodontics	**3**
Patient management	**7**
Oral diagnosis	**4**
Operative dentistry	**4**
Endodontics	**5**
Oral and maxillofacial surgery and pain control	**4**
Orthodontics and paediatric dentistry	**15**
Prosthodontics	**5**
Overall	**49**

**Table 4:** Numbers of questions that ChatGPT 3.5 answered correctly but ChatGPT 4.0 answered wrong for the Overseas Registration Examination (ORE) questions by dental subjects.

	No. of questions that ChatGPT 3.5 answered correctly but ChatGPT 4.0 answered wrong
Periodontics	**0**
Pharmacology and therapeutics	**4**
Oral medicine	**4**
Oral surgery	**3**
Oral pathology	**2**
Dental materials	**5**
Radiology	**3**
General dentistry	**4**
Child dental health and orthodontics	**18**
Restorative dentistry	**10**
Overall	**53**

## Discussion

This study demonstrated that GPT-4 could pass the 2 written dental licensing examinations, the US INBDE and the UK ORE, marking a significant milestone in the potential application of AI in patient management and dental education. This result suggests that GenAI has the potential to support dentists by providing correct oral health information to patients. The training of dental students and professionals may also need to be modified to accommodate the changes in patient needs^28^ and the impact of ChatGPT on knowledge acquisition, problem-solving, and decision-making. This necessitates reviews and reforms in dental education to adapt to the rapid changes in knowledge during the era of digital dentistry. Some dental knowledge may quickly become outdated. Moreover, both dental professionals and patients can now easily access fairly accurate dental knowledge through GenAI. As a result, dental professionals may not require extensive training in dental knowledge but instead need to focus on learning how to verify the accuracy of dental knowledge generated by the GenAI and apply it to individual patients. Consequently, the dental curriculum and assessment methods may undergo drastic changes, requiring different competencies and skill sets.

The multiple-choice questions adopted in this study covered various subjects of dental knowledge, such as oral surgery, orthodontics, periodontics, and prosthodontics, ensuring that the study's findings are relevant and applicable to daily practice. In addition, the GenAI system's performance in individual dental subjects was also assessed to allow for a more detailed understanding of the GenAI's strengths and weaknesses within various subjects of dental knowledge. Using multiple-choice questions provided an objective assessment of knowledge and avoided the potential error in analysing the content of answers.

This study adds to the growing evidence that GenAI systems can achieve humanlike performance in various knowledge subjects. Previous research has shown that GenAI models have been successful in several fields, such as medicine, business, and finance.^10^^,^^29^^,^^30^ This study extends these results to dentistry and demonstrates that GenAI can be a valuable tool in dental practice and education. In the context of professional licensure, the accuracy of oral health information of the latest generation of GenAI can be on par with human dental graduates.

Whilst this study presents the potential of GenAI in dentistry, the results should still be interpreted with caution. First, this study only adopted a single GenAI model, ChatGPT, and its performance may not be generalisable to other GenAI models. Changes may also be possible in future versions of the same GenAI model, such as the decrease in performance regarding child dental health and orthodontics in this study and the observations that GPT-4 answered some questions wrong whilst GPT-3.5 answered correctly. Second, the study focused on the multiple-choice written examinations, which could only comprehensively evaluate some aspects of dental knowledge. Multiple-choice answers may be correct, but this answer may be due to chance or wrong rationale. Moreover, the inability of ChatGPT to analyse figures or tables may also limit its potential use. Third, the sample of ORE and INBDE questions may only represent part of the spectrum of dental knowledge and skills, and GPT-4 may perform differently in other dental licensing examinations. To ensure reliable and accurate responses, the GenAI model needs to be refined for enhanced contextual understanding and developed to update its knowledge base seamlessly with the latest treatment protocols and guidelines.^31^^,^^32^

Future research could explore the performance of GenAI models in various aspects of dental subjects using subjective assessments other than multiple-choice examinations. As GenAI models like ChatGPT mature, investigations into the implementations of GenAI in other aspects of dentistry beyond diagnostics, screening, and treatment planning, such as automatic patient engagement, dental training, oral health education, and clinic management, would also be needed.^33^^,^^34^ Patient-reported outcomes on using AI in their management could also be investigated. The collection of big data, possibly at the population level, enables AI systems to interpret variations and provide more accurate disease diagnoses for individual patients. Once the disease model has been constructed through deep computational analysis, the prognosis and treatment outcomes can be simulated by computer (in silico), as opposed to taking place in a test tube (in vitro) or in a living organism (in vivo).^33^^,^^35^^,^^36^ These advancements may allow for a more personalised approach to dental management.^34^

## Conclusion

The latest generation GenAI, GPT-4, demonstrated proficiency in passing the dental licensing examinations and performed relatively well in various dental subjects. This has significant implications for integrating GenAI in health care and dental education delivery. However, further research is needed to explore the long-term impact of GenAI on dentistry and to address the challenges and barriers associated with its implementation. Clinicians and dental researchers should stay updated on the latest developments in GenAI and be aware of their potential impacts on their practice and research.

## Conflict of interest

None disclosed.
